# Is a weekly qualitative picket fence test sufficient? A proposed alternate EPID‐based weekly MLC QA program

**DOI:** 10.1002/acm2.13699

**Published:** 2022-07-20

**Authors:** Chaitanya Kalavagunta, Huijun Xu, Baoshe Zhang, Sina Mossahebi, Michael MacFarlane, Kai Jiang, Sung‐Woo Lee, Shifeng Chen, Amit Sawant, Arun Gopal, ByongYong Yi

**Affiliations:** ^1^ Department of Radiation Oncology University of Maryland School of Medicine Baltimore Maryland USA

**Keywords:** American Association of Physics in Medicine Task Group 142, electronic portal imaging device, failure mode and effects analysis, leaf‐end leakage, multileaf collimator quality assurance, picket fence, volumetric‐modulated arc therapy

## Abstract

**Purpose:**

Well‐designed routine multileaf collimator (MLC) quality assurance (QA) is important to assure external‐beam radiation treatment delivery accuracy. This study evaluates the clinical necessity of a comprehensive weekly (C‐Weekly) MLC QA program compared to the American Association of Physics in Medicinerecommended weekly picket fence test (PF‐Weekly), based on our seven‐year experience with weekly MLC QA.

**Methods:**

The C‐Weekly MLC QA program used in this study includes 5 tests to analyze: (1) absolute MLC leaf position; (2) interdigitation MLC leaf position; (3) picket fence MLC leaf positions at static gantry angle; (4) minimum leaf‐gap setting; and (5) volumetric‐modulated arc therapy delivery. A total of 20,226 QA images from 16,855 tests (3,371 tests × 5) for 11 linacs at 5 photon clinical sites from May 2014 to June 2021 were analyzed. Failure mode and effects analysis was performed with 5 failure modes related to the 5 tests. For each failure mode, a risk probability number (RPN) was calculated for a C‐Weekly and a PF‐Weekly MLC QA program. The probability of occurrence was evaluated from statistical analyses of the C‐Weekly MLC QA.

**Results:**

The total number of failures for these 16,855 tests was 143 (0.9%): 39 (27.3%) for absolute MLC leaf position, 13 (9.1%) for interdigitation position, 9 (6.3%) for static gantry picket fence, 2 (1.4%) for minimum leaf‐gap setting, and 80 (55.9%) for VMAT delivery. RPN scores for PF‐Weekly MLC QA ranged from 60 to 192 and from 48 to 96 for C‐Weekly MLC QA.

**Conclusion:**

RPNs for the 5 failure modes of MLC QA tests were quantitatively determined and analyzed. A comprehensive weekly MLC QA is imperative to lower the RPNs of the 5 failure modes to the desired level (<125); those from the PF‐Weekly MLC QA program were found to be higher (>125). This supports the clinical necessity for comprehensive weekly MLC QA.

## 1 INTRODUCTION

The multileaf collimator (MLC) is an essential component for modern treatment delivery techniques like intensity‐modulated radiation therapy (IMRT) and volumetric‐modulated arc therapy (VMAT).^1^ Geometric verification of MLCs is essential to assure treatment accuracy.^2^ The increased use of hypofractionated radiation therapy has resulted in a higher dose per fraction. Due to the lower number of fractions, the impact of uncaught errors per fraction is more severe. Average time elapsed between weekly picket fence (PF‐Weekly) and monthly MLC test is 15 days. Thus, the probability of an MLC error going undetected is higher in a hypofractionated course. Rangel et al.^3^ showed that a 1‐mm error in MLC position results in 2.7% and 5.6% changes in the equivalent uniform dose of clinical target volumes in prostate and head‐and‐neck plans, respectively. This would justify MLC quality assurance (QA) tests for both VMAT‐ and non‐split IMRT‐based treatments and has placed increased importance on the adoption of an efficient and appropriate routine MLC QA program. One of the drawbacks with measurement‐based IMRT‐verification QA or patient‐specific QA is that its accuracy is guaranteed only at the moment of the measurement.^4^ This approach may be inadequate for multi‐machine facilities with matching beam energies and the same MLC configurations, where patients can be transferred between different machines. A periodic comprehensive MLC QA program is thus required to guarantee the delivery of treatments as planned. The need for comprehensive MLC QA becomes even more crucial in verifying the consistency of MLC characteristics when using calculation‐based verification QA (e.g., commercial software, including Mobius3D^5^ [Varian Medical Systems; Palo Alto, CA], MU Check^6^ [Oncology Data Systems. Inc.; Oklahoma City, OK], RadCalc^7^ [LAP Laser; Lüneburg, Germany], Diamond^8^ [PTW; Freiburg, Germany], or IMSure^9^ [Standard Imaging, Inc.; Middleton, WI]) to ensure that the modeled MLC characteristics do not change with time.^10^ The desired MLC QA should also accommodate current clinical IMRT treatment techniques (step‐and‐shoot or sliding‐window IMRT and VMAT).

The American Association of Physicists in Medicine (AAPM) TG‐142 report^11^ recommends a qualitative picket fence test on a weekly basis. On a monthly basis, the report recommends tests verifying setting versus radiation field (non‐IMRT) for two patterns, travel speed (IMRT), and leaf position accuracy (IMRT). However, to our knowledge, the rationale behind the choice of frequency and type of test has not been described.

In 2017, AAPM published MPPG 8.a for linac performance tests,^12^ stating: “The purpose of this guideline was to provide a list of critical performance tests in order to assist the qualified medical physicist in establishing and maintaining a safe and effective QA program.” Based on risk assessment performed on tests from AAPM Task Group reports on linac QA, this practice guideline highlighted those tests that were effective at maintaining quality and safety for the patient. The guideline also compared the risk probability numbers (RPNs) of daily and monthly TG142 tests to those from O'Daniel's^13^ failure modes and effect analysis (FMEA) approach to TG142. The guideline shows an average RPN score of 101 and normalized RPN scores of 100 in Appendix I^12^ (Table I Weekly tests). However, no supporting data explain how these scores were obtained. MPPG 8.a also recommended using a PF‐Weekly test.^12^

A literature review of vendor specifications and AAPM Task Group reports on MLC position tolerances shows a ±1 mm for accuracy and ±0.5 mm for reproducibility tests. For example, Varian specifies a tolerance of ±1 and ±0.5 mm for MLC leaf‐end position accuracy and reproducibility, respectively, for both the Millennium 120 and HD120 MLC models used in the Trilogy, TrueBeam, and TrueBeam Edge machines.^14^, ^15^, ^16^, ^17^ Varian‐recommended tolerances for MLC positional accuracy tests in the TrueBeam Multi Performance Check (MPC) self‐check tool are as follows: maximum and mean leaf offset: ±1 mm, and maximum and mean leaf reproducibility: ±0.5 mm.^18^ The AAPM recommends tolerances of ±1 mm^11^ (TG‐142, monthly leaf position accuracy test [IMRT]) and ±0.5 mm (TG‐50).^19^ During a beam delivery of IMRT/VMAT plans, if the difference between the actual leaf position and the planned one is larger than the set tolerance, the Varian linac triggers an MLC interlock, which invokes a “beam hold‐off.” For Varian machines, factory‐set defaults for dose dynamic leaf tolerance for sliding window, minimum segment size, or VMAT are 2, 1, and 5 mm, respectively, and can be changed in Aria Treatment Administration.

To the best of our knowledge, the rationale behind the AAPM‐suggested^11^, ^12^ PF‐Weekly test has not been justified by a study. The aim of this work is to present the comprehensive weekly (C‐Weekly) MLC QA program and compare it with AAPM TG‐142 and MPPG 8.a recommended PF‐Weekly test to ascertain whether the recommended test type and frequency are sufficient for a sensitive detection of MLC deviation from baseline.

In 2016, the AAPM published Task Group Report 100 (TG‐100), which contained a methodology for a risk analysis–based quality management for radiation therapy using an FMEA approach.^20^ FMEA can be used as an objective tool to compare two processes by identifying the process with the higher risk of failure. MPPG 8.a recommends a weekly qualitative picket fence test for the MLC with an average RPN score of 101.^12^ In this study, FMEA was used to validate the C‐Weekly MLC QA program by comparing its RPN values with those of PF‐Weekly MLC QA.

**FIGURE 1:**
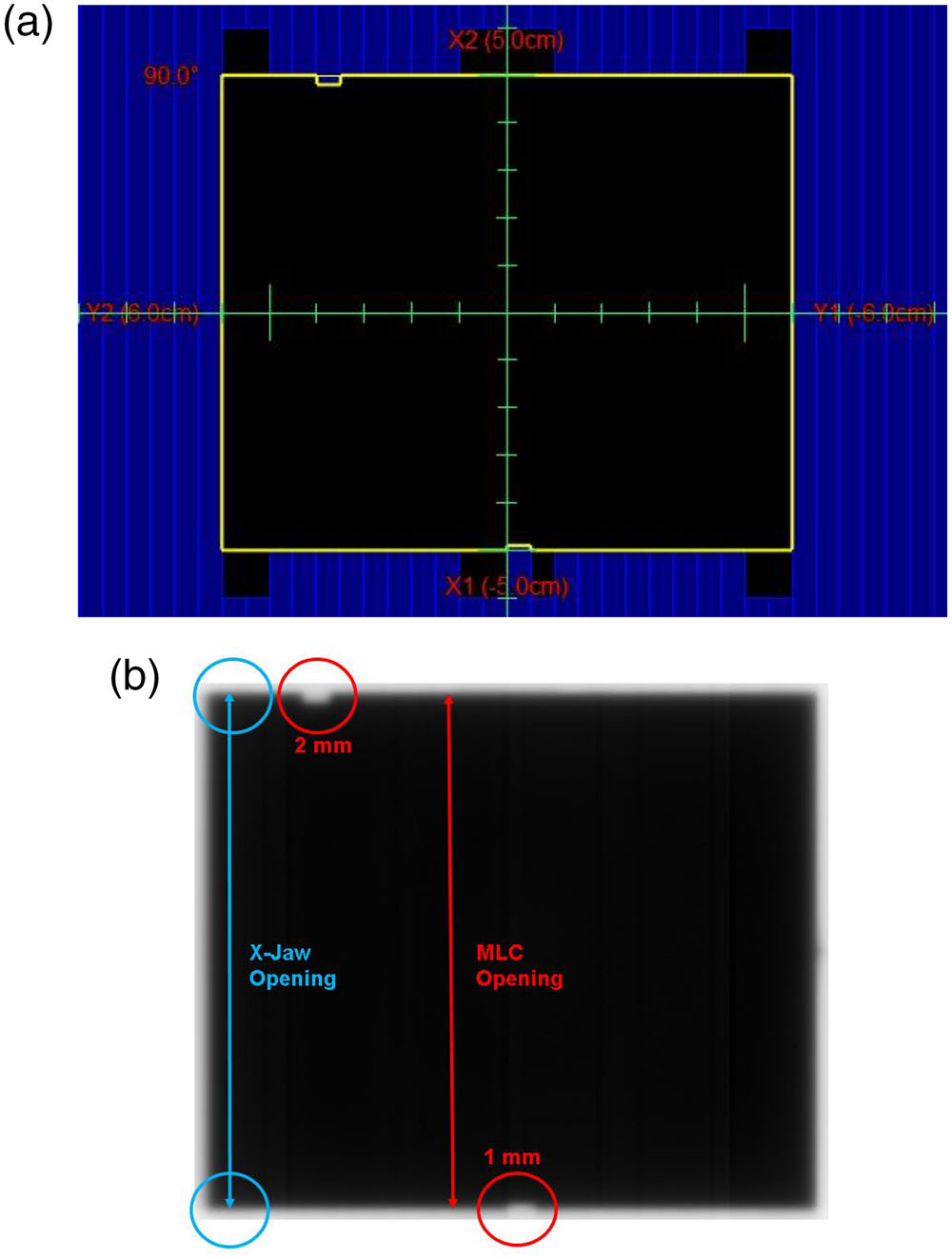
Absolute MLC leaf position test (a) field setup. (b) EPID analysis image. Blue circles = jaw opening positions; red circles = MLC positions.

**FIGURE 2:**
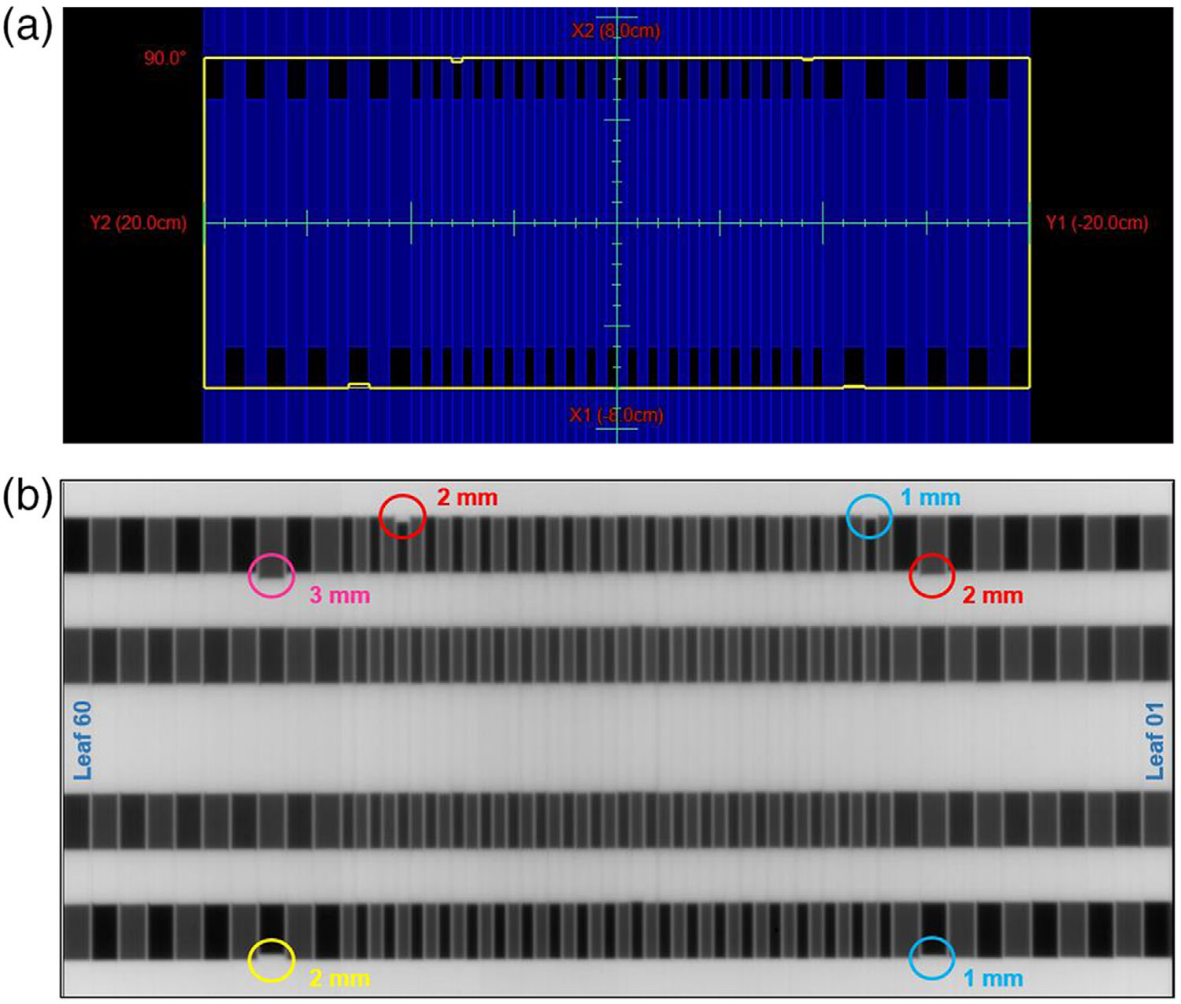
Interdigitation leaf position test (a) field setup. (b) EPID analysis image with intentional off MLC positions: yellow circle = 2 mm; pink circle = 3 mm; red circles = 2 mm; blue circles = 1 mm.

**FIGURE 3:**
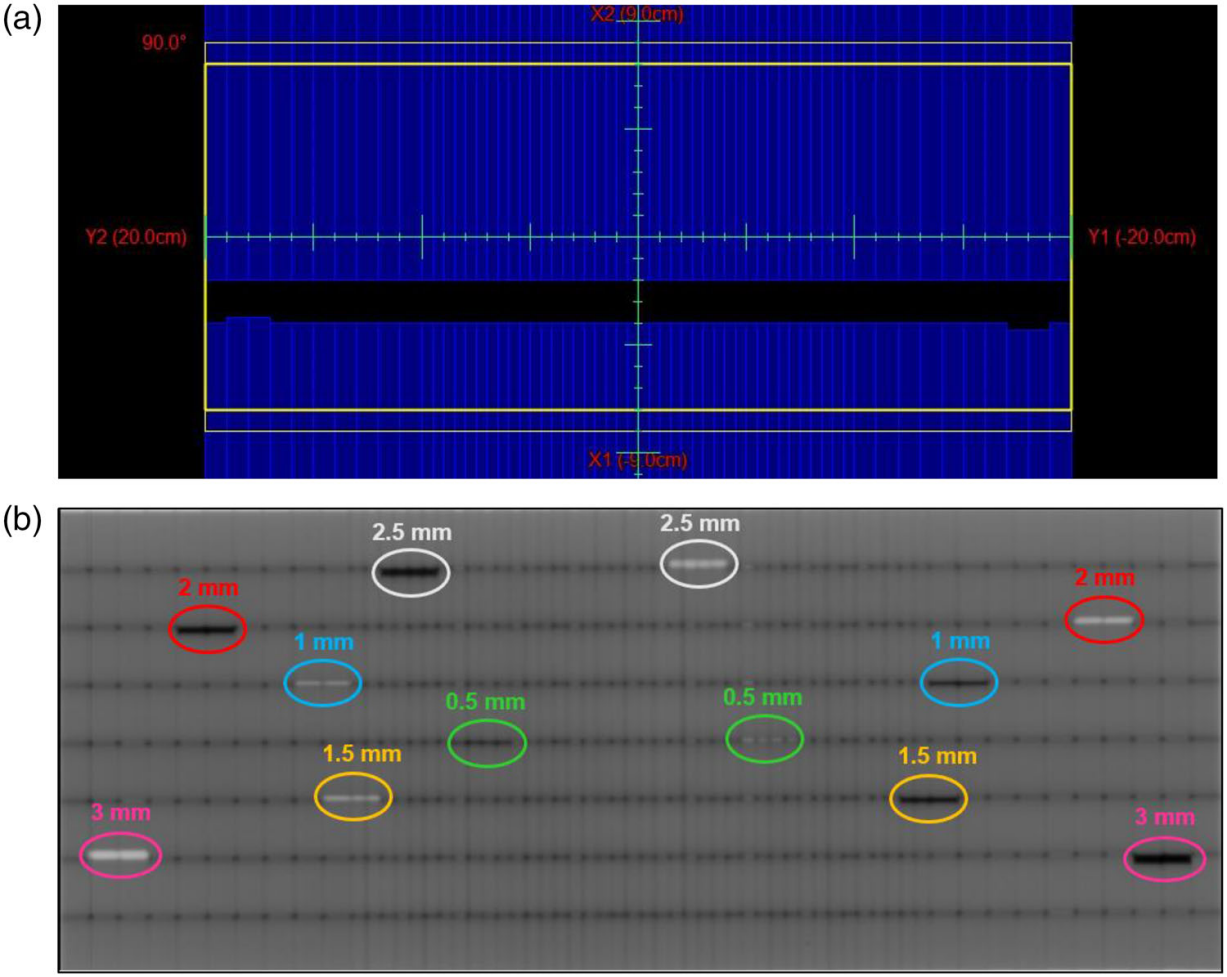
Static gantry picket fence test (a) field setup. (b) EPID analysis image. White and black colors show intentional overrun and underrun off‐positions, respectively. Intentionally off MLC positions: pink circles = 3 mm; red circles = 2 mm; blue circles = 1 mm; yellow circles = 1.5 mm; white circles = 2.5 mm; and green circles = 0.5 mm.

**FIGURE 4:**
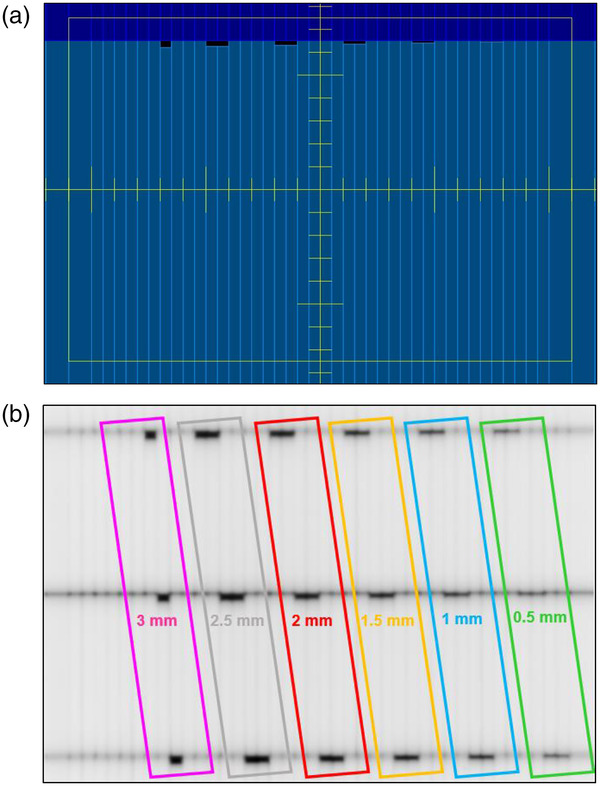
Minimum leaf gap setting test (a) field setup. (b) EPID analysis image. Intentionally off MLC positions: pink = 3 mm; gray = 2.5 mm; red = 2 mm; yellow = 1.5 mm; blue = 1 mm; and green = 0.5 mm.

**FIGURE 5:**
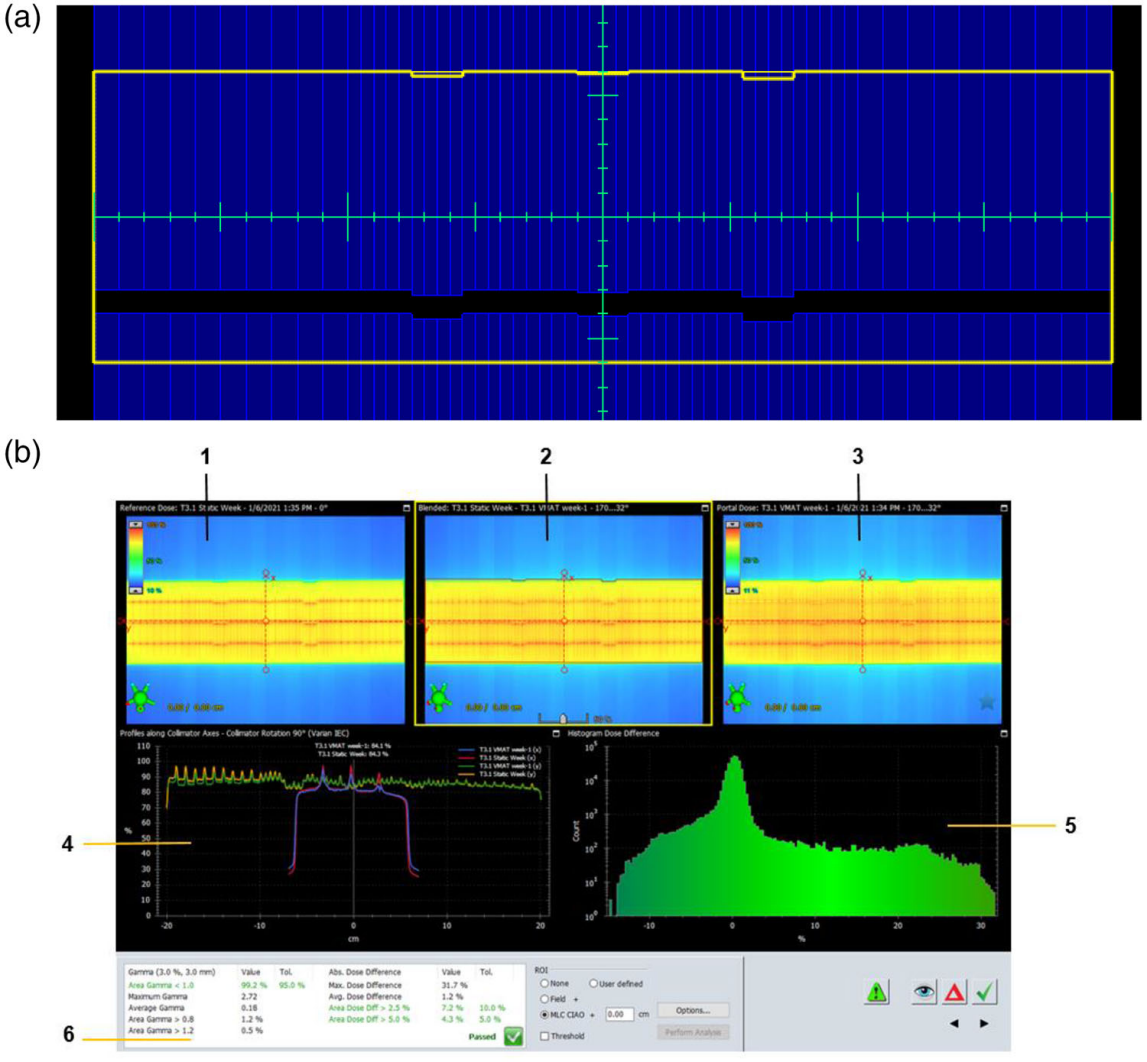
VMAT test (a) field setup. (b) EPID analysis image using the ARIA‐integrated Portal Dosimetry application. Panes 1, 3, and 2 show portal dose images of fields from the sliding‐window intensity‐modulated radiation therapy (IMRT) plan, VMAT plan, and blended overlay of both plans, respectively. Pane 4 shows dose profiles along specified collimator axes. Pane 5 shows the histogram of the dose differences. Pane 6 shows gamma analysis results.

In this study, we propose a C‐Weekly MLC QA plan consisting of six beams that test the motion ranges of MLCs and leaf banks during interdigitation, picket fence, minimum leaf gap setting, and VMAT motion. The tests can be performed in 10 min and analyzed in 5 min. Figures 1, 2, 3, 4, 5 show electronic portal imaging device (EPID)‐based images from these five tests. The MLC patterns in tests 1–4 show intentional offset positions of 0.5–2 mm for visual verification. Machine parameters, test resolution, and analysis parameters are shown in Table 1. The test resolution and analysis parameters were chosen based on AAPM TG‐142^11^ and TG‐119^21^ reports, SRS‐based considerations, and clinical experience.

**TABLE 1:** Machine parameters and analysis criteria of weekly multileaf collimator (MLC) tests

No.	Test	Criteria*
1	**Absolute MLC Leaf Position**	
	Jaw opening^m^	±0.2 cm^b^
	MLC opening^m^	±0.2 cm^b^
	Jaw‐MLC alignment^m^	0.1 cm^b^
2	**Interdigitation Leaf Position**	
	Beam delivery^a^	Image visibility
	Leaf position^m^	0.1 cm^b^
	Offset position precision^m^	0.1 cm^b^
3	**Static Gantry Picket Fence**	
	Leaf position^m^	0.1 cm^b^
	Minimum size of visible offset position^m^	0.05 cm^d^
4	**Minimum Leaf Gap Setting**	
	Leaf position^m^	0.1 cm^b^
	Minimum size of visible offset position^m^	0.05 cm^d^
5	**VMAT**	
	Area of 2.5% dose difference^a^	10%^e^
	Area of 5% dose difference^a^	5%^e^
	Area gamma <1.0 (3% 3 mm)^a^	95%^c^
	EPID alignment in *X* and *Y* direction^a^	0.05 cm^e^

*Note*: Gantry angle: tests 1–4: 0°, test 5 VMAT beam: Start: 172°, End: 32°. Collimator angle: 90°. EPID position: fixed. SID: 100 cm. *Minimum offset position for image visibility or analysis criteria are shown with superscript “m” or “a” respectively. Justifications for test parameters are shown using superscripts b, c, d, and e, where b: TG‐142^11^, c: TG‐119^21^, d: SRS considerations, and e: clinical experience.

Abbreviations: EPID, electronic portal imaging device; MLC, multileaf collimator; VMAT, volumetric‐modulated arc therapy.

## 2 MATERIALS AND METHODS

Tests were performed on 11 Varian linear accelerators (7 C‐series, 3 TrueBeam, and 1 TrueBeam‐Edge) at 5 photon clinical sites: 1 university clinic with 4 linacs, 3 community sites with 2 linacs, and 1 community site with 1 linac at the time of data acquisition. Among the 11 linacs, 3 were installed during the period of data collection, 1 was decommissioned, and 1 was taken over by another hospital system. Two MLC systems were tested: HD120, with leaf sizes of 2.5 and 5 mm; and Millennium 120, with leaf sizes of 5 and 10 mm. A 100‐cm source–imager distance (SID) was chosen based on the largest MLC field size. For each test, the collimator was rotated to 90° to align the longer dimension of the portal imager along the MLC bank. Although these MLC patterns can be collected by either films or an EPID imager, all images in this study were collected using Varian EPID aS1000 and aS1200 imager models. The aS1000 has a resolution of 1024 × 768 pixels and an active area of 40.1 × 30.1 cm^2^ (equivalent to 0.392‐mm pixel size).^22^ The aS1200 has a resolution of 1280 × 1280 pixels and an active area of 43.1 × 43.1 cm^2^ (equivalent to 0.336‐mm pixel size).^23^

### 2.1 Tests

#### 2.1.1 Absolute MLC leaf position

This test is for absolute leaf positions obtained by analyzing a portal image of a radiation field formed by jaws and MLCs. The positions of leaves can be determined from the known positions of the jaws. Figure 1a,b shows the field setup and EPID output used for analysis. Two MLC leaves have been placed at intentional off‐positions, which are moved into the field by 1 and 2 mm with respect to the rest. This test and the following tests can be evaluated visually at the machine or with the help of Varian ARIA software Offline Review or Portal Dosimetry applications (Varian Medical Systems; Palo Alto, CA).

#### 2.1.2 Interdigitation leaf position

This test checks interdigitation leaf positions as a result of motion by analyzing a portal image made by capturing the composite of four interdigitated fields. An interdigitation pattern was chosen as this is a hard motion condition that would introduce friction between the leaves. Figure 2a,b shows the field setup and EPID output used for analysis. Three MLC leaves have been intentionally moved to off‐positions of 1, 2, and 3 mm with respect to the rest.

#### 2.1.3 Static gantry picket fence

This is a variation of the picket fence test with intentional leaf offset positions. The picket fence is a quick visual test that can check the reproducibility of the leaves’ positions and results compared with baseline where a “picket” is the line formed by several MLC pairs all at the same position with a 1‐mm gap between the MLC strips. Figure 3a,b shows the field setup and EPID output used for analysis. The differences between our test and the regular picket fence test are four MLC leaves that are designed to intentionally overrun and underrun off‐positions of 0.5, 1, 1.5, 2, 2.5, and 3 mm with respect to the rest.

#### 2.1.4 Minimum leaf gap setting

This test examines the consistency of the minimum leaf gap setting with intentional offset leaf‐end leakage positions, which is essential for VMAT plans and non‐split IMRT motion of large‐field‐size IMRT plans. Figure 4a,b shows the field setup and EPID output used for analysis, respectively. There are 18 MLC leaves that have been intentionally moved to offset positions of 0.5, 1, 1.5, 2, 2.5, and 3 mm with respect to the rest. The aim of the minimum leaf gap setting test is a reproducibility check and therefore it should be pointed out that although the smallest leaf gap setting tested in the C‐Weekly program was 0.5 mm, this value could be different for other institutions.

#### 2.1.5 VMAT test

This test focuses on the delivery and dosimetry of VMAT beams. This is done by performing a gamma analysis of a VMAT beam with a sliding‐window beam the same MUs for each control point. Figure 5a,b shows the field setup and EPID output used for the analysis. Twelve MLC leaves have been intentionally moved to off‐positions of 1–2 mm with respect to the rest. Unlike the VMAT beam, the sliding‐window beam has a fixed gantry angle and dose rate. The pattern used here was based on RapidArc QA tests recommended by Varian.^24^, ^25^

### 2.2 Evaluation of FMEA

Weekly MLC tests for 368 cumulative weeks (16 855 tests) from 11 machines from May 2014 to June 2021 were analyzed. Failure modes were identified by ways in which the MLC might fail. Possible consequences of these failures were identified. The FMEA of the C‐Weekly program was conducted as described in AAPM TG‐100.^20^ Probability of occurrence (*O*), likelihood of detection (*D*), and severity of the effect (*S*) of failure modes were scored from 1 to 10 based on weekly MLC QA experience and RPN scoring criteria.^26^ Table 3 shows the lookup table used to generate the *O*, *S*, and *D* values. The percentage of occurrences was calculated using the formula, $\mathrm{Occurance}\;(\%)\;=\frac{\mathrm{total}\;\mathrm{no}.\;\mathrm{of}\;\mathrm{fails}}{\mathrm{total}\;\mathrm{no}.\mathrm{of}\;\mathrm{tests}}\;\;\times 100$. This number was converted to *O* value using the lookup Table 3. The detectability of the monthly QA was determined to be 4 per expert assessment. As the frequency of weekly MLC QA was four to five times that of monthly MLC QA, the probability of detecting failures for weekly will be increased by the same amount. The four to five times higher probability decreased the detectability scale by 2 as shown in Table 4. The detectability values of C‐Weekly's static gantry picket fence and PF‐Weekly's picket fence were set the same, as they are essentially the same test. The severity values were assigned using criteria from Sawant et al.^26^ as shown in Table 2. The *S* and *D* values were obtained using the lookup Table 3. The risk for each failure mode was assigned an RPN = *O* × *S* × *D*, such that the RPN values ranged from 1 (low risk, 1 × 1 × 1) to 1000 (high risk, 10 × 10 × 10). For each failure mode, RPN was calculated for the C‐Weekly and PF‐Weekly programs. A TG‐100 established corrective action threshold of RPN <125 was used for this study.^20^

**TABLE 2:** Severity values using criteria from Sawant et al.^26^ (Used with permission)

**Value**	**Severity of effect** **^a^**
1	No adverse event (AE)
2‐3	Grade 1: mild; asymptomatic or mild symptoms; clinical or diagnostic observations only; intervention not Indicated
4‐5	Grade 2: moderate; minimal, local or noninvasive intervention indicated; limiting age‐appropriate instrumental activities of daily living (ADL)^b^
6‐7	Grade 3: severe or medically significant but not immediately life‐threatening; hospitalization or prolongation of hospitalization indicated; disabling; limiting self‐care ADL^c^
8‐9	Grade 4: life‐threatening consequences. Urgent intervention indicated
10	Grade 5: patient death related to AE

^a^ *Source*: National Cancer Institute Common Terminology Criteria for Adverse Events v4.0 (2009).

^b^Instrumental ADL: preparing meals, shopping for groceries or clothes, using the telephone, managing money, and so on.

^c^Self‐care ADL: bathing, dressing and undressing, feeding self, using the toilet, taking medications, and not bedridden.

**TABLE 3:** Scale used in assigning probability of occurrence (*O*), severity of effect (*S*), and detectability (probability of failure to detect, *D*) values

Value	Occurrence (%)	Severity	Detectability (%)
1	0.01	No AE	0.0
2	0.02	Grade 1	0.2
3	0.04	Grade 1	0.5
4	0.05	Grade 2	1.0
5	0.40	Grade 2	2.0
6	0.50	Grade 3	5.0
7	1.00	Grade 3	10.0
8	2.00	Grade 4	15.0
9	5.00	Grade 4	20.0
10	>5	Grade 5	25.0

AE: Adverse Event.

## 3 RESULTS

Weekly MLC QA tests were successfully performed for 98% of the targeted weeks. The total number of failures for tests was 143 (0.9%): 39 (27.3%) for the absolute MLC position test, 13 (9.1%) for the interdigitation position test, 9 (6.3%) for static gantry picket fence, 2 (1.4%) for the minimum leaf gap setting test, and 80 (55.9%) for the VMAT motion test. Nine fails were picked up by the static gantry picket fence test that amounts to 6.3% of all fails. These results do not include fails attributed to the portal imager rather than MLC issues. Five failure modes resulting from erroneous MLC bank and/or leaf movement corresponding to the individual tests were identified: (1) static field treatment MLC deviation from expected (DFE) position; (2) undeliverable beam due to interdigitation motion; (3) step‐and‐shoot IMRT delivery MLC (DFE) position; (4) leaf‐end leakage; and (5) VMAT delivery. The effects of each failure mode could in general lead to an underdose or overdose. In test 1 (absolute MLC leaf position) a DFE would result in a smaller/larger MLC opening leading to an underdose/overdose. A test 2 (interdigitation leaf position) fail would result in an undeliverable beam that would result in possible treatment delay/stop. A test 3 (step‐and‐shoot IMRT delivery) fail would lead to an overdose/underdose. This is especially important when using small beamlets and field edge matching using MLCs with up to a 20% discrepancy at the match edge.^27^, ^28^, ^29^ In test 4 (minimum‐leaf gap setting), leaf‐end leakage would result if the leaf end distance is larger than expected, and we will have an overdose. On the other hand, a smaller than expected leaf‐end would lead to an underdose. There is a chance of MLC leaf collision that will delay treatment. In test 5 (VMAT), the expected dose would not be delivered as intended if the number of MU for a certain gantry position and MLC position do not match. Table 4 shows the failure modes, the effect of these failures, and calculated RPN scores. The RPN scores of the failure modes for the PF‐Weekly program ranged from 60 to 192, higher than desired (RPN < 125) for some of the tests. In contrast, the RPN scores for the C‐Weekly program were in the range of 48–96 (all <125).

**TABLE 4:** Failure modes and risk probability number (RPN) scores for the comprehensive weekly (C‐Weekly) and weekly picket fence (PF‐Weekly) programs

		C‐Weekly				PF‐Weekly			
Failure mode	Effect of failure	*O*	*S*	*D*	RPN	*O*	*S*	*D*	RPN
Static field MLC position deviation	Under/overdose	7	6	2	84	7	6	4	168
Interdigitation: beam not deliverable	Possible treatment delay/stop	5	5	2	50	5	5	4	100
Step‐and‐shoot IMRT delivery: MLC position deviation	Under/overdose	5	6	2	60	5	6	2	60
Leaf‐end leakage	Under/overdose	4	6	2	48	4	6	4	96
Dose difference between VMAT and static IMRT with same control points	Under/overdose	8	6	2	96	8	6	4	192

## 4 DISCUSSION

The use of an FMEA approach used in this study comes with its own uncertainties and weaknesses. FMEA is a subjective analysis and is therefore highly user dependent. There is a chance of underestimating the risk if all failure modes are not considered.^30^ An FMEA approach may not be suitable if a system has several failures at once as it does not consider the correlation between them.^30^ The approach depends on the exhaustive testing of the detectability pathways. If a problem is not detected, then it cannot be identified by FMEA.

Each test in the C‐Weekly program has different levels of intended offset positions in the delivery patterns. A visual verification of these positions confirms the accuracy of the test. Inability to find any off‐positions other than those expected indicates that no MLC leaves are off by more than 0.5 mm. This tighter margin is especially useful for the small‐field stereotactic radiosurgery program using HD120 for treating trigeminal cases with a 4–5 mm‐beam aperture that is sensitive to MLC positioning. Therefore, although the 0.5‐mm‐test tolerance used in this program is much tighter than some recommended by vendors and the AAPM, it is still within vendor‐suggested MLC operational specifications, and a 0.5‐mm‐tolerance‐related test failure should not be identified as an “error” or suggest that the MLC is not performing as expected.

For each test, the rotation of the collimator to 90° to align the longer dimension of the portal imager along the MLC bank is an absolute requirement only for the aS1000 EPID imager model. This rotation was chosen for all the tests to deliver the same plan across machines and less dependent on EPID model and to invoke maximum gravity constraint.

The AAPM‐recommended^11^, ^12^ PF‐Weekly test^31^ is a quick visual test to check the reproducibility of leaf positions and compare the results with baseline. This test is insufficient, because it does not capture all the expected failure patterns demonstrated in this study, such as determination of the absolute leaf position, minimum leaf gap setting, VMAT delivery issues, motion range of MLCs, and effects of gravity and challenging motion conditions like interleaf interaction during interdigitation.

The C‐Weekly program is scheduled for each machine and delivered by therapists in the middle of the week. Use of EPID for C‐Weekly program makes it practically possible in the busy clinic. EPID has been considered a good tool for testing MLC position verification and for dynamic dose evaluation as it has high resolution and fast response time.^32^, ^33^, ^34^, ^35^, ^36^ It does not require a tool to install, and no special setup is needed but to open the EPID and then dry run the programmed beams. There are a total of six beams for five tests. Beam delivery takes approximately 10 min per machine, and the analysis can be accomplished within 5 min by a medical physicist or a medical physics resident using Varian Portal Dosimetry software. This allows reasonable time for delivery and analysis and is thus easily implementable. The clinical burdens of the PF‐Weekly versus the C‐Weekly program are not much different, because access to the machines in a busy clinic is the main issue and not time spent on delivery or analysis. As long as an institution follows TG‐142, no additional costs should be associated with the implementation of the C‐Weekly program. A reduction of the RPN to the desired level can be expected.

The QA compliance rate was 98.25%, where the compliance rate is given by the following formula: $\mathrm{compliance}\;\mathrm{rate}=\frac{\mathrm{total}\;\mathrm{no}.\;\mathrm{of}\;\mathrm{scheduled}\;\mathrm{tests}-\mathrm{noncompliances}}{\mathrm{total}\;\mathrm{no}.\;\mathrm{of}\;\mathrm{scheduled}\;\mathrm{tests}}$ × 100. During the study period, 289 tests were not analyzed because of wrong EPID imager positions or partial beam delivery, resulting from either EPID imager issues (maintenance, incorrect imager position [e.g., SID = 150 cm instead of 100 cm]) or plan delivery interruption leading to partial imaging, which cannot be used for the analysis. After excluding these tests, the effective compliance rate became 100%. Thus, this program is practical for busy clinics, regardless of clinic size.

The tests themselves are qualitative with a level of quantitative verification. Each test checks the geometric limitation, which is also the verification power of the test. These tests are basically reproducibility tests but are also quantitative. The suggested C‐Weekly program is designed for conformal therapy, step‐and‐shoot IMRT, and VMAT. One limitation of this program is that the five beams do not explicitly test sliding‐window IMRT. One method of testing sliding‐window IMRT is to add a step‐and‐shoot delivery to the plan and compare the acquired images to those obtained with sliding‐window and VMAT delivery. Such a test is in the process of being added to our comprehensive MLC QA program. This was done by adding a step‐and‐shoot delivery to the plan and acquiring the image. The VMAT and sliding‐window IMRT delivery images were then compared to the step‐and‐shoot images.

## 5 CONCLUSIONS

A C‐Weekly MLC QA program has been designed and tested. The FMEA approach of AAPM‐recommended PF‐Weekly‐only MLC QA shows that it failed to determine some failure modes. The RPN scores of the MLC QA test failure modes have been quantitatively and objectively determined based on the statistical analysis in this study. The newly generated RPN scores for the PF‐Weekly program are >125 for most of the failure modes, whereas those of the C‐Weekly program are <125. It has been demonstrated that a weekly picket fence test as recommended in TG‐142 is not sufficient. A C‐Weekly MLC QA is therefore recommended to fulfill the goal of QA, while not appreciably increasing the clinical burden in terms of delivery and the analysis.

## CONFLICT OF INTEREST

The authors declare that there is no conflict of interest.

## AUTHOR CONTRIBUTION

The authors confirm their contribution to the paper as follows: study conception and design: Chaitanya Kalavagunta, ByongYong Yi; data acquisition: Chaitanya Kalavagunta, Huijun Xu, Sina Mossahebi, Michael MacFarlane, Kai Jiang; analysis and interpretation of results: Chaitanya Kalavagunta, ByongYong Yi; draft manuscript preparation: Chaitanya Kalavagunta, Huijun Xu, Baoshe Zhang, Sina Mossahebi, Michael MacFarlane, Kai Jiang, Sung‐Woo Lee, Shifeng Chen, Amit Sawant, Arun Gopal, ByongYong Yi; final approval of the version to be published: Chaitanya Kalavagunta, Huijun Xu, Baoshe Zhang, Sina Mossahebi, Michael MacFarlane, Kai Jiang, Sung‐Woo Lee, Shifeng Chen, Amit Sawant, Arun Gopal, ByongYong Yi. All authors reviewed the results and approved the final version of the manuscript.

## Data Availability

Data available on request from the authors.
